# Analysis of Facial Skeletal Morphology: Nasal Bone, Maxilla, and Mandible

**DOI:** 10.1155/2021/5599949

**Published:** 2021-05-24

**Authors:** Han-Sheng Chen, Szu-Yu Hsiao, Kun-Tsung Lee

**Affiliations:** ^1^Dental Department, Kaohsiung Municipal Siao-gang Hospital, Kaohsiung, Taiwan; ^2^School of Dental Medicine, Kaohsiung Medical University, Kaohsiung, Taiwan; ^3^Department of Dentistry for Child and Special Needs, Kaohsiung Medical University Hospital, Kaohsiung, Taiwan; ^4^Division of Clinical Dentistry, Department of Dentistry, Kaohsiung Medical University Hospital, Kaohsiung, Taiwan; ^5^Department of Oral Hygiene, College of Dental Science, Kaohsiung Medical University, Kaohsiung, Taiwan

## Abstract

The growth and development of facial bones are closely related to each other. The present study investigated the differences in the nasomaxillary and mandibular morphology among different skeletal patterns. Cephalograms of 240 participants were divided into 3 groups based on the skeletal pattern (Class I, Class II, and Class III). The dimensions of nasomaxilla (nasal bone length, nasal ridge length, nasal depth, palatal length, and maxillary height) and mandible (condylar length, ramus length, body length, symphysis length, and entire mandibular length) were measured. One-way analysis of variance and Pearson's correlation test were used for statistical analysis. No significant differences were observed among the skeletal patterns in terms of nasal bone length, palatal length, maxillary height, or condylar length. Class II had a significantly shorter ramus, mandibular body, and entire mandibular length compared with those of Class I and Class III. Nasal ridge length exhibited a significant moderate correlated with nasal bone length (correlation coefficient: 0.433) and maxillary height (correlation coefficient: 0.535). The entire mandibular length exhibited a significant moderate correlated with ramus length (correlation coefficient: 0.485) and body length (correlation coefficient: 0.536). In conclusion, nasal and maxillary dimensions exhibited no significant difference among the 3 skeletal patterns. Mandibular body and entire mandibular lengths were significantly positively correlations with Class III skeletal patterns.

## 1. Introduction

Craniofacial development is regulated by dynamic and complex mechanisms that involve various signaling cascades and gene regulation pathways [1]. Manlove et al. [2] concluded that the development of the craniofacial skeleton occurs as a result of a sequence of normal developmental events in the brain, the optic pathway, speech and swallowing function, the pharyngeal airway, muscles, and teeth. The growth and development of facial bones are closely related processes. The nasomaxillary complex comprises numerous bones that articulate with each other at sutures. The frontal bone and ethmoid bone meet the maxilla on both sides and protrude from the upper-middle portion of the face. The upper third of the nose is supported by the nasal bones that articulate with the frontal bone at the superior border and with the frontal process of the maxilla at the lateral border. The lower two-thirds of the nose is supported by the lateral nasal cartilage [3]. In addition, bone resorption at the inner surface of the nasomaxillary complex enlarges the maxillary sinus. Thus, nasomaxillary growth and development occur by bone apposition, bone resorption, and remodeling. [4–6]

Growth centers of the mandible include the mandibular body, mandibular angle, condylar process, coronoid process, symphysis, and alveolar process. Growth of the condylar process takes place from its tip to the mandibular canal and foramen and is affected by the position of the petrous portion of the temporal bone. Growth of the mandibular condylar cartilage increases the mandibular ramus length, entire mandible length, and the bilateral condylar distance. Additionally, the development of dentition and alveolar bone growth also increases the mandibular body length. Enlow [5] noted that the forward-downward growth of the maxilla and mandible has an expanding V configuration and defined such growth pattern as relocation. The extent and direction of bone growth varies among individuals. Changes in the pattern and rate of bone growth can lead to abnormal bone morphology and malocclusion. The present study investigated the parameters of nasomaxillary and mandibular bone morphologies and their correlations. The null hypothesis was that no difference exists in the nasal, maxillary, or mandibular dimensions among different skeletal patterns.

## 2. Materials and Methods

Cephalograms of 240 individuals (120 male and 120 female) were obtained for this study. Participants were selected on the basis of the availability of cephalograms and whether they were 18 to 39 years old. The cephalograms were divided into 3 groups according to the skeletal pattern based on the A point–nasion–B point (ANB) angle as follows: Class I malocclusion (0° < ANB < 4°), Class II malocclusion (ANB ≥ 4°), and Class III malocclusion (ANB < 0°). Each group consisted of 80 participants (40 males and 40 females). The following participants were excluded from the study: (1) those with a pathologic disease in the facial bone, (2) those that had undergone craniofacial surgery, and (3) those with a history of maxillofacial trauma.

**Figure 1:**
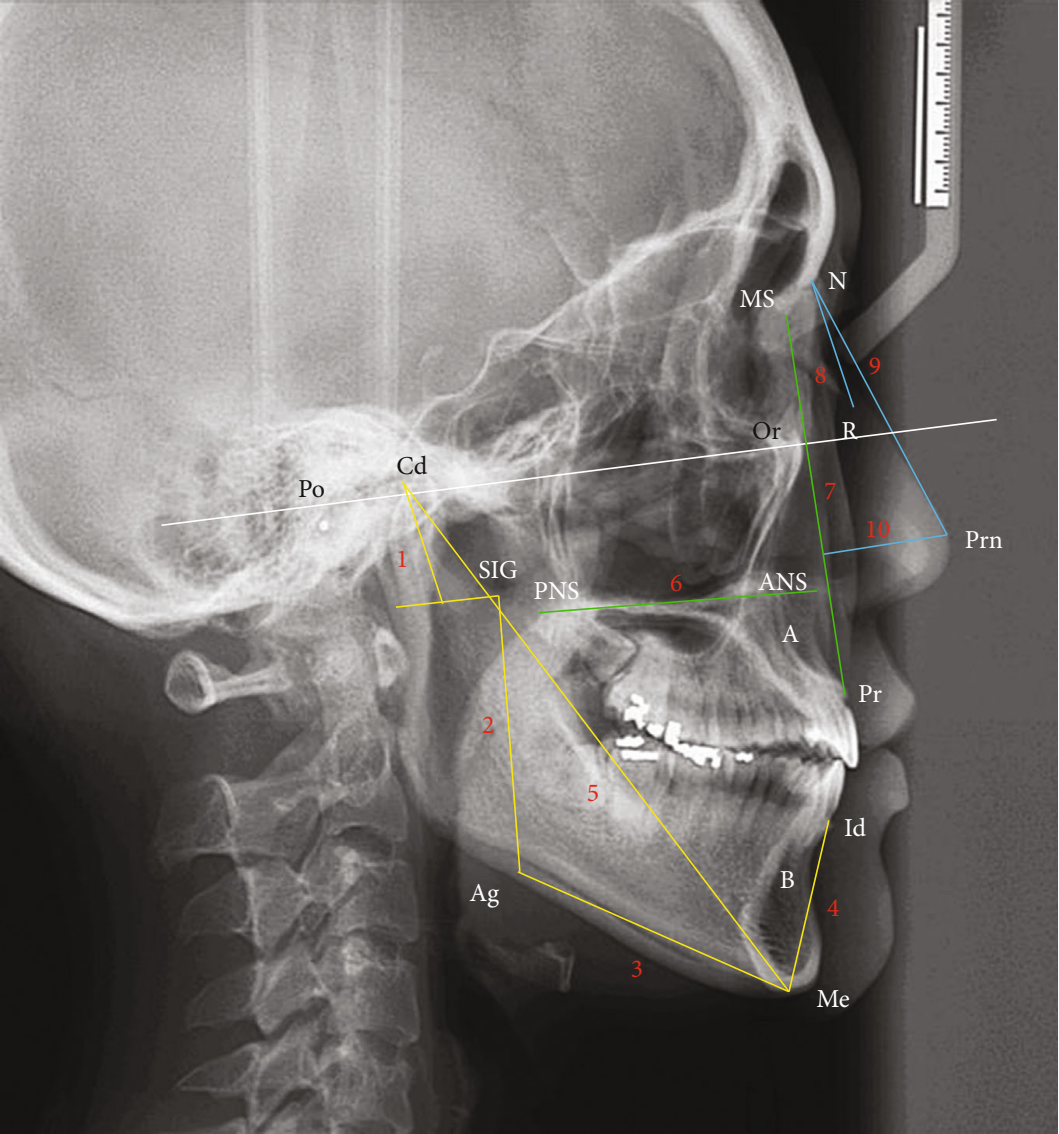
Cephalometric landmarks and linear measurements. N: nasion; Or: orbitale; Po: porion; R: rhinion; MS: frontomaxillary nasal suture; Prn: pronasale; ANS: anterior nasal spine; point A; PNS: posterior nasal spine; Pr: prosthion; Id: infradentale; point B; Cd: condylion; Ag: antegonial notch; SIG: sigmoid notch; Me: menton. Yellow line (mandible): 1: condylar length; 2: ramus length; 3: body length; 4: symphysis length; 5: entire length. Green line (Maxilla): 6: palatal length; 7: maxillary height. Blue line (nose): 8: nasal bone length; 9: nasal bridge length; 10: nasal depth.

We followed the methods proposed by Hsiao et al. [7] in 2020. The following landmarks (Figure 1) were identified on the cephalogram: nasion (N); orbitale (Or); porion (Po); rhinion (R); the most anterior and inferior point on the tip of the nasal bone; frontomaxillary nasal suture (MS); the superior-most point of the suture where the maxilla articulates with the frontal and nasal bone; pronasale (Prn); anterior nasal spine (ANS); point A; posterior nasal spine (PNS); prosthion (Pr); infradentale (Id); point B; condylion (Cd); antegonial notch (Ag); sigmoid notch (SIG); and menton (Me). Nasal dimensions were calculated according to the nasal bone length (N to R), nasal ridge length (N to Prn), and nasal depth (Prn vertical to the MS-Pr line). Maxillary dimensions were calculated according to the palatal length (ANS to PNS) and maxillary height (MS to Pr). Mandibular dimensions were calculated according to the condylar length (the longest distance from Cd to a line parallel to Or-Po line through SIG), ramus length (SIG to Ag), body length (Ag to Me), symphysis length (Me to Id), and entire length (Cd to Me). Regarding the measurement error of our cephalometric study, the intraclass correlation coefficient (0.982) was >0.9, thus confirming consistency in the repeated measurements.

Data were analyzed using SPSS version 20 (IBM, Armonk, NY, USA). Intragroup and intergroup comparisons were performed using Student's *t*-test and one-way analysis of variance, respectively. Post hoc comparisons were performed using Tukey's honestly significant difference test. Pearson's correlation test was used to compare correlations among the variables in each group. We describe the correlation strength for the absolute value of the ratio as follows: very weak (0-0.19), weak (0.20-0.39), moderate (0.40-0.59), strong (0.60-0.79), and very strong (0.80-1.0). A *P* value < 0.05 was considered statistically significant. This retrospective study was approved by a human investigation review committee (KMUHIRB-E(II)-20180200).

## 3. Results

Table 1 presents the results of the analysis of the 3 skeletal patterns (Class I, Class II, and Class III). Intergroup comparison revealed no significant correlation between age and skeletal pattern (*P* = .216). Furthermore, no significant difference in nasal bone length, nasal ridge length, nasal depth, palatal length, or maxillary height was noted among the skeletal patterns. Intergroup comparisons of condylar length and symphysis length among the 3 skeletal patterns revealed no significant differences. However, the patients in Class II had a significantly shorter ramus length (52.8 mm), mandibular body length (59.8 mm), and entire mandibular length (117.9 mm) than those of the patients in Class I (55.7, 62.4, and 125.1 mm, respectively) and Class III (55.8, 66.1, and 131.7 mm, respectively). Therefore, the null hypothesis was accepted for the nasal and maxillary morphology and rejected for the mandibular morphology.

As indicated in Table 2, no significant intergroup differences were observed in the nasomaxillary, condylar, or symphysis lengths among male patients. However, among the male patients, those in Class II had significantly shorter mandibular ramus, body, and entire lengths than those in Class III. Among the female patients, those in Class II had greater maxillary lengths than those in Class III (Table 3). Analysis of all skeletal patterns revealed no significant difference among female patients in terms of condylar and symphysis lengths. However, female patients in Class II had a significantly shorter ramus length than those in Class I and a significantly shorter mandibular body and entire mandible length than those in Class III.

**Figure 2:**
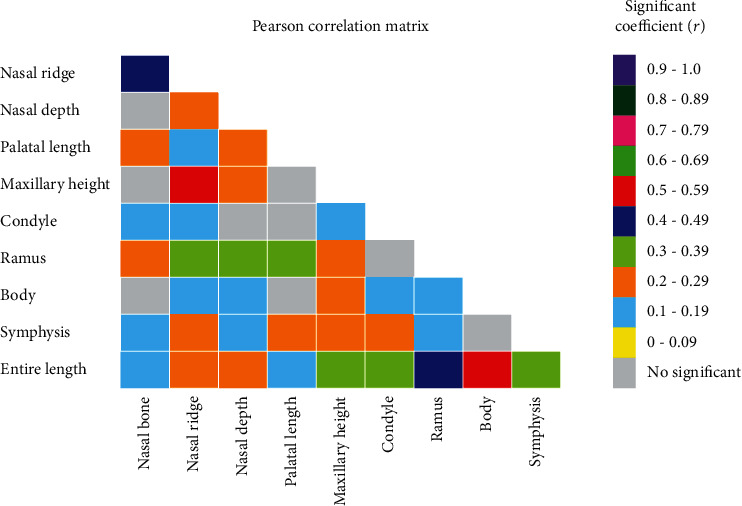
Pearson correlation matrix. Absolute value of correlation ratio: very weak (0-0.19), weak (0.20-0.39), moderate (0.40-0.59), strong (0.60-0.79), and very strong (0.80-1.0).

Table 4 lists the nasomaxillary and mandibular lengths for each skeletal classification compared using Pearson's correlation coefficient. Age exhibited no significant correlation with maxillary or mandibular lengths. The mandibular body length (correlation coefficient: 0.279) and entire length (correlation coefficient: 0.236) exhibited a significant positive correlation with skeletal classification; for example, an individual with a Class III skeletal pattern had a longer mandibular body and entire lengths. A significant negative correlation was noted between variations of the ANB angle and the mandibular body length (correlation coefficient: -0.524) and entire mandibular length (correlation coefficient: -0.544). A highly significant positive correlation was observed between maxillary height and ridge length (correlation coefficient: 0.535). The Pearson correlation matrix was shown in Figure 2.

The condylar length had significant positive correlations with the mandibular body, symphysis, entire mandibular, maxillary, nasal bone, and nasal ridge lengths. Ramus length exhibited no correlation with condylar length but had a significant positive correlation with all other variables. Mandibular body length was not correlated with symphysis length or nasal bone length but was significantly correlated to all other variables. The entire mandibular length had a significant positive correlation with all variables.

## 4. Discussion

Facial profile pattern is closely associated with nasal development. Heijden et al. [8] noted that the growth rate of the nose is related to body height; that is, the nose develops as the height increases. They also indicated that the nose reaches its maximum growth rate between the age of 10 and 11 years in the female population and between 12 and 13 years in the male population [8]. Posen [9] reported that 90% of nasal bone development is usually completed by the age of 13 years, at which age male and female nasal bone growth patterns are fundamentally similar. Heijden et al. [8] also reported that 95% of nasal bones have developed by the age of 16 and 15 years in male and female populations, respectively. Posen [9] reported that 91% of nasal ridge (length of the dorsum of the external nose) growth is completed by the age of 16 years, and the development takes longer in male patients than in female patients; however, the difference is not statistically significant.

Nasal depth begins to increase by the age of 6 months, with growth in the nasal cartilage accounting for much of the increase in nasal depth. In general, the nasal depth stops increasing by the age of 15 years, although it could continue in some cases until the age of 17–18 years. According to Posen [9], more time is required for the growth for each part of the male nose compared with that required for the growth of the female nose. At equivalent ages, nasal development in females is more mature than in males, but no significant sex difference exists. On the basis of the aforementioned research, the present study selected participates aged >18 years because the nose has nearly or completely developed by this age. According to our findings, there were no significant differences in nasal dimensions in relation to skeletal patterns. Therefore, the null hypothesis was accepted for the nasal morphology. According to Pearson's correlation analysis of age, no significant difference existed among the nasomaxillary and mandibular dimensions. Thus, it can be concluded that facial bone development had probably stabilized at the age of 18 years. Hwang et al. [10] reported that nasal bone length was not significantly different between Korean male and female populations. Park et al. [11] also reported no significant intersex difference in the nasal septum and external nose growth processes. In our study, nasal bridge length and nasal depth were significantly greater in male patients than in female patients. However, nasal bone length was not significantly different between male and female patients.

Nehra [12] also revealed that nasal length and nasal depth are not significantly correlated with the sella-nasion to A point (SNA) angles. Our results also revealed that nasal ridge length and nasal depth were not significantly correlated with ANB angle. However, a significant negative correlation with nasal bone length was observed upon examining the ANB angles. A negative ANB angle results in a long nasal bone. Therefore, the Class III skeletal pattern had a longer nasal bone than the Class II pattern. Park et al. [11] reported that nasal bone growth is significantly correlated with nasal ridge length and nasal depth throughout an individual's life. Moreover, Nehra [12] reported the existence of a significant positive correlation between the nasal ridge length and nasal depth; similar results were observed in our study. Moreover, we found that nasal bone length was significantly and moderately correlated (correlation coefficient: 0.433) with nasal ridge length.

We found the nasal bone length was significantly and positively correlated with palatal length, indicating that when the palatal increases in length and extends forward, the maxilla is elongated, which in turn causes the nasal bone to grow forward. However, our study also revealed that the nasal bone length was not significantly correlated with maxillary height. This may be because only a smaller portion of the maxillary and nasal bones are connected on both sides, leading to weaker effects on growth potency. Nehra [12] concluded that nasal ridge length has a significant positive correlation with upper anterior facial height and palatal length, which implies that the anteroposterior length of the maxilla strongly affects nasal ridge length; the same study also reported a significant positive correlation between nasal depth and upper anterior facial height. Our study showed a similar result; there was a significant moderate correlation (0.535) between the nasal ridge length and maxillary height. In our study, Pearson's correlation test revealed that compared with palatal length, maxillary height had a significant positive correlation with the development of each mandibular part. Thus, maxillary height develops at a similar rate as mandibular length, and both may begin to increase in late adolescence. All these findings allow us to make inferences regarding the relationship between palatal length, maxillary height, skeletal pattern, and occlusal condition. An increase in maxillary height is induced by alveolar bone development and tooth eruption and occurs later than an increase in palatal length.

Regarding maxillary development, Nanda et al. [13] suggested that maxillary growth over the age of 12 years is small, with <1° change to the SNA angle. Nahhas et al. [14] studied the growth and development of the maxilla and found that maxillary growth onset, peak, and cessation occurred relatively later in the male population than in the female population. Peak growth occurred at the age of 10.8 years in girls and 13.4 years in boys, by the age of 16 years, over 90% of maxillary development and growth had ceased in both male and female populations. Nahhas et al. [14] also showed that palatal lengths are greater in the male population than in the female population. In the present study, we found that the palatal length was significantly greater in male patients than in female patients for both Class II and Class III skeletal patterns; this might be the result of abnormal occlusion induced by the overgrowth of the maxilla and mandible during late adolescence. Because development in the male population occurs during late adolescence, men have a greater maxillary height than women in all skeletal patterns. Our study identified no significant differences in palatal length or maxillary height among any of the skeletal patterns. Therefore, the null hypothesis was accepted for the maxillary morphology.

Gomes and Lima [15] showed that no significant difference in mandibular development exists between the sexes or Class I and II skeletal patterns. Bjork [16] reported that during the adolescent growth spurt, condylar growth peaked at the age of approximately 14.5 years. The condylar growth rate is greater during adolescence than during childhood and in male than in female populations. With regard to mandibular growth, our study revealed that condylar length was not significantly different for the various skeletal patterns; Class II and III male patients had a significantly greater condylar length than those of female patients. This result is consistent with that observed for palatal length, which means that an increase in condylar length in both male and female populations is necessary for skeletal formation with Class I features. In other words, Class I skeletal patterns are related to maxillary palatal and mandibular condylar lengths.

Gomes and Lima [15] reported that the ramus length in Class I and II skeletal patterns has no correlation with mandibular development or sex. Compared with the lengths of other mandibular structures (i.e., mandible body and entire mandibular lengths), ramus length has the least variability in annual growth rate. Our study showed that the ramus length in Class II was significantly shorter than that in other classes. Therefore, ramus length is a key factor for identifying the Class II skeletal pattern. Growth of the ramus in Class II may be complete at an earlier stage than that in Class I and Class III. Therefore, ramus length in Class I and III is substantially greater than that in Class II. Unlike Gomes and Lima [15], we noted that the ramus length is significantly greater in the male population than in the female population for all skeletal patterns.

Singer et al. [17] and Lambrechts et al. [18] suggested that the depth of the antegonial notch can be used to predict the potential and direction of mandibular growth. Singer et al. [17] stated that patients with a deep mandibular notch have a more retrusive mandible, shorter mandibular body, and shorter ramus height. These patients also have a longer total facial height, longer lower facial height, and less mandibular growth than patients with a shallow notch, indicating that the chin bone does not shift forward. We agree with these studies [17, 18] and believe that the antegonial notch is an essential growth center. Muscular movements affect the mandibular growth process through functional shaping and reinforcement [4–6]. The antegonial notch is the attachment site of the masseter and medial pterygoid muscles; hence, it is strongly affected by muscular movements. On the basis of the physiology of mandibular bone development, our study used the antegonial notch as the separation growth point for the mandibular ramus and body.

Bae et al. [19] reported that the growth of the mandibular body peaks between the ages of 13 and 15 years, and the extent of growth is remarkably greater in the male population than in the female population. Gomes and Lima [15] noted no significant differences in mandibular body length between Class I and Class II skeletal patterns. In our study, we noted the longest mandibular body length in the Class III skeletal patterns and the shortest in the Class II pattern. Aki et al. [20] estimated the pattern and size of the mandibular symphysis to predict the direction and potential of mandibular growth and concluded that symphysis length and depth increase with age, and their growth rates increase during puberty. The growth spurts of symphysis length and depth occur during and after adolescence, with a greater increase in symphysis length than in symphysis depth, although the increases are less pronounced in the female population than in the male population. Ricketts [21] proposed that symphysis morphology could be used to predict the direction of mandibular growth. Our study revealed that symphysis development exhibited no significant difference in all skeletal patterns.

Although the growth of each mandibular structure often has a significant positive correlation with that of another, condylar growth is negatively correlated with ramus growth. This negative correlation, although not significant, suggests that the ramus tends to be short when the condyle is long. The entire mandibular length has a significant positive correlation with the growth of each mandibular structure and with the length of nasal and maxillary structures. These results indicate a close relationship between the growth and development of facial bones. Moreover, the entire mandibular length was significantly and moderately correlated with the mandibular ramus (correlation coefficient: 0.485) and body (correlation coefficient: 0.536) lengths.

## 5. Conclusion

Nasal measurements (nasal bone length, nasal ridge length, and nasal depth) exhibit no correlation with skeletal patterns. Palatal length and maxillary length, which represent maxillary development, have no correlation with skeletal patterns. Neither condylar length nor symphysis lengths are correlated with skeletal patterns. Notably, Class III has the greatest ramus, mandibular body, and entire mandibular lengths, whereas Class II has the shortest ramus, mandibular body, and entire mandibular lengths. The present study provides a comprehensive understanding of the relationships among the nasal bone, maxilla, and mandible. Our findings may be useful to physicians for the analysis of craniofacial development and selection of appropriate treatment plans. However, the main limitation of this study is that it involved two-dimensional cephalometric analysis, but comparisons were performed among the participants' actual three-dimensional anatomical features.

## Figures and Tables

**Table 1:** Patients characteristics in the skeletal classification (one-way ANOVA).

Variables	Class I (F/M = 40/40)			Class II (F/M = 40/40)			Class III (F/M = 40/40)					Intergroup comparison
	Mean	SD		Mean	SD		Mean	SD		F		Significant
Age	24.4	4.71	─	24.7	4.76	─	23.5	4.48	─	1.540	─	
ANB	2.1	0.93	─	6.3	1.90	─	-3.6	2.97	─	447.404	^∗^	Class II > Class I > Class III
Nasomaxillary												
Nasal bone length	28.1	4.00	─	27.7	2.95	─	28.4	3.82	─	0.775	─	
Nasal ridge length	57.8	5.73	^†^	58.5	3.71	^†^	58.5	4.67	^†^	0.510	─	
Nasal depth	28.2	4.54	^†^	27.7	3.35	^†^	28.0	3.04	^†^	0.305	─	
Palatal length	52.2	3.86	─	52.8	3.50	^†^	51.5	3.69	^†^	2.302	─	
Maxillary height	73.6	7.82	^†^	74.9	4.64	^†^	73.5	5.71	^†^	1.317	─	

Mandible												
Condylar length	21.2	2.91	─	21.1	3.27	^†^	22.0	3.50	^†^	2.034	─	
Ramus length	55.7	5.56	^†^	52.8	5.43	^†^	55.8	6.09	^†^	7.552	^∗^	Class III > Class II, Class I > Class II
Body length	62.4	5.43	─	59.8	4.00	─	66.1	4.91	^†^	34.855	^∗^	Class III > Class I > Class II
Symphysis length	36.4	5.51	─	36.5	4.00	^†^	36.2	4.54	^†^	0.095	─	
Entire length	125.1	6.81	^†^	117.9	12.96	─	131.7	9.23	^†^	38.378	^∗^	Class III > Class I > Class II

F: Female; M: Male. ^†^: intergender comparison (M > F): statistically significant, *P* < 0.05; ^∗^: intergroup comparison: statistically significant, *P* < 0.05; ─: not significant.

**Table 2:** The characteristics of male patients in the skeletal classification (one-way ANOVA).

Variables	Class I (*n* = 40)		Class II (*n* = 40)		Class III (*n* = 40)				Intergroup comparison
	Mean	SD	Mean	SD	Mean	SD	F		Significant
Age	24.8	4.38	24.7	5.23	23.2	4.27	1.413	─	
ANB	2.0	0.96	6.0	1.62	-4.1	2.89	257.542	^∗^	Class II > Class I > Class III
Nasomaxillary									
Nasal bone length	27.8	3.20	27.9	2.94	29.2	3.78	2.225	─	
Nasal ridge length	59.4	6.65	59.9	3.84	60.6	3.81	0.538	─	
Nasal depth	30.3	4.96	29.0	3.80	29.2	2.59	1.387	─	
Palatal length	52.8	3.31	54.4	3.31	53.2	3.34	2.674	─	
Maxillary height	75.5	5.48	76.0	5.41	76.7	4.64	0.535	─	

Mandible									
Condylar length	21.4	2.86	21.9	3.70	22.8	2.85	2.255	─	
Ramus length	57.5	5.28	55.4	5.00	59.2	5.87	4.979	^∗^	Class III > Class II
Body length	62.7	4.50	59.5	3.65	68.1	4.33	43.447	^∗^	Class III > Class I > Class II
Symphysis length	37.2	6.41	38.3	4.11	38.7	3.90	1.016	─	
Entire length	127.5	7.03	120.0	17.08	137.4	6.88	23.586	^∗^	Class III > Class I > Class II

*n*: number of patient. ^∗^: statistically significant, *P* < 0.05; ─: not significant.

**Table 3:** The characteristics of female patients in the skeletal classification (one-way ANOVA).

Variables	Class I (*n* = 40)		Class II (*n* = 40)		Class III (*n* = 40)				Intergroup comparison
	Mean	SD	Mean	SD	Mean	SD	F		Significant
Age	24.0	5.05	24.7	4.31	23.7	4.73	0.4	─	
ANB	2.2	0.90	6.6	2.12	-3.2	3.02	199.9	^∗^	Class II > Class I > Class III
Nasomaxillary									
Nasal bone length	28.3	4.69	27.5	2.98	27.6	3.74	0.5	─	
Nasal ridge length	56.2	4.11	57.1	3.03	56.4	4.56	0.5	─	
Nasal depth	26.1	2.81	26.5	2.28	26.7	2.97	0.7	─	
Palatal length	51.6	4.30	51.1	2.88	49.9	3.28	2.6	─	
Maxillary height	71.6	9.28	73.8	3.45	70.2	4.79	3.3	^∗^	Class II > Class III
Mandible									
Condylar length	21.1	2.99	20.3	2.59	21.2	3.92	1.1	─	
Ramus length	54.0	5.36	50.2	4.55	52.5	4.23	6.8	^∗^	Class I > Class II
Body length	62.1	6.27	60.1	4.36	64.1	4.66	6.1	^∗^	Class III > Class II
Symphysis length	35.6	4.37	34.7	3.02	33.6	3.63	2.8	─	
Entire length	122.8	5.77	115.8	6.26	126.0	7.66	25.1	^∗^	Class III > Class II

*n*: number of patient. ^∗^: statistically significant, *P* < 0.05; ─: not significant.

**Table 4:** Pearson's correlation coefficient test in the nasomaxillary and mandibular dimensions.

	Age	Skeletal pattern	ANB	Nasal bone	Nasal ridge	Nasal depth	Palatal length	Maxillary height	Condyle	Ramus	Body	Symphysis	Entire length
Nasomaxillary length													
Palatal length	0.084	-0.074	0.178^∗^	0.237^∗^	0.164^∗^	0.204^∗^	1	0.016	0.062	0.340^∗^	-0.029	0.292^∗^	0.149^∗^
Maxillary height	-0.115	-0.007	0.07	0.096	0.535^∗^	0.298^∗^	0.016	1	0.188^∗^	0.279^∗^	0.293^∗^	0.272^∗^	0.325^∗^
Nasal bone	-0.099	0.04	-0.135^∗^	1	0.433^∗^	0.117	0.237^∗^	0.096	0.151^∗^	0.245^∗^	-0.032	0.175^∗^	0.168^∗^
Nasal ridge	-0.011	0.057	-0.044	0.433^∗^	1	0.248^∗^	0.164^∗^	0.535^∗^	0.189^∗^	0.311^∗^	0.166^∗^	0.223^∗^	0.288^∗^
Nasal depth	0.063	-0.024	-0.047	0.117	0.248^∗^	1	0.204^∗^	0.298^∗^	0.089	0.331^∗^	0.151^∗^	0.140^∗^	0.291^∗^

Mandibular length													
Condyle	-0.034	0.098	-0.175^∗^	0.151^∗^	0.189^∗^	0.089	0.062	0.188^∗^	1	-0.073	0.134^∗^	0.220^∗^	0.303^∗^
Ramus	0.017	0.007	-0.267^∗^	0.245^∗^	0.311^∗^	0.331^∗^	0.340^∗^	0.279^∗^	-0.073	1	0.157^∗^	0.293^∗^	0.485^∗^
Body	-0.103	0.279^∗^	-0.524^∗^	-0.032	0.166^∗^	0.151^∗^	-0.029	0.293^∗^	0.134^∗^	0.157^∗^	1	0.114	0.536^∗^
Symphysis	-0.008	-0.019	0.02	0.175^∗^	0.223^∗^	0.140^∗^	0.292^∗^	0.272^∗^	0.220^∗^	0.293^∗^	0.114	1	0.378^∗^
Entire length	-0.08	0.236^∗^	-0.544^∗^	0.168^∗^	0.288^∗^	0.291^∗^	0.149^∗^	0.325^∗^	0.303^∗^	0.485^∗^	0.536^∗^	0.378^∗^	1

^∗^: Statistically significant, *P* < 0.05.

## Data Availability

The data used to support the findings of this study are available from the corresponding author upon request.
